# Waist-to-Height Ratio as a Key Predictor for Diabetes and Hypertension in Lao PDR National Health Survey

**DOI:** 10.1177/10105395241295573

**Published:** 2024-10-31

**Authors:** Kethmany Ratsavong, D. R. Essink, Manithong Vonglokham, Sengchanh Kounnavong, Somphou Sayasone, Wichai Aekplakorn, Suchin Worawichawong, E. P. Wright

**Affiliations:** 1 Lao Tropical and Public Health Institute, Vientiane Capital, Lao PDR; 2 Athena Institute, Vrije University Amsterdam, Amsterdam, The Netherlands; 3 Department of Community Medicine, Faculty of Medicine Ramathibodi Hospital, Mahidol University, Bangkok, Thailand; 4 Department of Pathology, Faculty of Medicine Ramathibodi Hospital, Mahidol University, Bangkok, Thailand; 5 Guelph International Health Consulting, Amsterdam, The Netherlands

**Keywords:** NCD screening, BMI, alternative noninvasive measures, WHtR, WC, Lao PDR

## Abstract

This study aimed to determine the potential predictive value of four noninvasive anthropometric indices in screening for the risk of diabetes and hypertension in the Lao population. The data used for this study were collected as part of the National Health Survey which used the World Health Organization’s stepwise approach, covered 17 provinces and Vientiane capital, and had a representative sample of 3240 participants above 18 years old. Among the anthropometry indices tested, waist-to-height ratio (WHtR) had the highest predictive power for the prevalence of diabetes (area under the curve [AUC] = 0.73) and hypertension (AUC = 0.70). It is suitable for use in urban or rural areas and for fieldwork. The WHtR can serve as a public health and clinical screening tool, as there are no differences between sexes, ages, and ethnicities when monitoring diabetes and hypertension risk in Lao PDR, using the optimal cutoff point of 0.5 for both diabetes and hypertension.

## What We Already Know

- Prevalence of diabetes and hypertension is high, with many unaware of disease status.
- Body mass index (BMI) is not a good indicator of obesity; nutrition research and policy are still inconsistent in the use of BMI as the obesity index.
- Other studies suggest that local or at least Asian cutoff values should be used.

## What This Article Adds

- This is the first study to report the performance of the waist-to-height ratio (WHtR) in the Lao population; among obesity indices, it had the highest predictive power for the prevalence of diabetes and hypertension.
- Although the Lao population includes many ethnic groups, the WHtR was equally useful in different ethnic groups.
- We could show that there was no gender difference in the usefulness of the WHtR as an indicator of obesity and related risk of NTDs.
- WHtR can be measured using a flexible measuring tape in both urban and rural areas.
- A cutoff value of 0.5 has been shown to be effective predictor in detecting risk of diabetes and hypertension.
- The cutoff value can provide a clear call-to-action message such as, “A healthy waist is less than half your height, so get moving and eat right”.

## Introduction

The highest prevalence and mortality of non-communicable diseases (NCDs) are found in low- and middle-income countries, including Lao PDR.^1^ The top ten causes of mortality in Lao PDR include stroke, which increased by 19.5% between 2009 and 2019, and diabetes, which increased by 30% in the same period.^2^ Stroke and heart disease are both influenced by hypertension (HTA), diabetes (DM), and other lifestyle and behavioral risk factors.^2^ In a recent Thai study, half of the patients with diabetes had HTA, whereas 14% of hypertensive patients had DM; 80% were unaware of their disease status. A similar situation prevailed in Lao PDR: only 42% of patients were aware of their DM and 29% of their HTA status.^1,3^ The co-existence of these two diseases is thought to increase the risk of cardiovascular disease. Overweight, especially abdominal obesity, is a strong risk factor for both DM and HTA.^4^ It is useful to be able to identify persons at risk for these potentially serious conditions.

In Lao PDR, overweight and obesity are usually (but not consistently) measured using body mass index (BMI), despite evidence that BMI is not always a good indicator of obesity, and that it may need different definitions in certain populations.^5^ Asian studies have recommended lowering the BMI cutoff that defines obesity, to be different from the standard used for Caucasians.^6,7^ Alternatives to BMI include waist circumference (WC) and waist-to-hip ratio (WHR), a simple obesity index for public health screening; a meta-analysis, mainly in Asian countries, suggested that waist-to-height ratio (WHtR) could be a simple noninvasive tool for identifying obesity and therefore cardiometabolic and DM risk with unisex cutoff.^8-11^

Identifying noninvasive indicators to screen for DM and HTA risk in low-resource settings is a priority topic in the Lao PDR health research agenda.^12^ Research should inform strategies to prevent and control NCDs in both communities and hospitalized patients.^13^ Information on suitable indicators is limited in Lao PDR. This study aimed to determine the predictive potential of noninvasive anthropometric indices: BMI, WC, HC, WHR, WHtR, and weight (with appropriate cutoff points for best indices and the standard indices), to screen for risk of DM and HTA in Lao PDR, to identify which should be recommended for future use in Lao PDR.

## Methods

### Study Area and Population

The data were collected as part of a national health survey, comprising a series of cross-sectional population surveys to track health status and risk factors and to provide information on the prevalence of NCDs such as DM and HTA in a representative sample of the Lao population. The survey covered 17 provinces and the capital city Vientiane, and included 3240 participants aged above 18 years, with proportional representation of males and females and urban and rural residents.

### Study Design

A cross-sectional study was implemented between March and August 2020 (details in Supplementary material, Annex 1: Sample Size Calculation, Annex 2: Sampling Procedures, Annex 3: Field and Laboratory Procedures).

## Data Collection

The six-member team of data collectors (nurses or other health workers) was trained to use the World Health Organization’s (WHO) stepwise approach^1^: (1) structured face-to-face interviews including socio-demographic questions; (2) physical measurements—blood pressure (BP), height, weight, and waist and hip circumferences; (3) biochemical measurements—fasting blood glucose (FBG) and HbA_1c_. Participants signed a consent form before interviews; they returned the next day for biochemical examinations, after fasting for 8 to 10 hours.

## Definition of Variables

Standardized WHO definitions and cutoff values for variables have been used. See (Text Box 1) for more information.

**Text Box 1.** Study Variables.

BMI: BMI was calculated according to WHO recommendation based on the formula body weight (kg) divided by body height (m)^2^. The cutoff values were defined as: - WHO BMI Standard cutoff^14^: ○ Underweight: BMI < 18.5 kg/m^2^, ○ Normal weight: BMI ≥ 18.5-24.9 kg/m^2^, ○ Overweight: BMI ≥ 25-29.9 kg/m^2^, ○ Obese: BMI ≥ 30 kg/m^2^ - BMI Asia-Pacific cutoff^1^: ○ Underweight: BMI < 18.5 kg/m^2^, ○ Normal weight: BMI ≥ 18.5 to 22.9 kg/m^2^, ○ Overweight: BMI ≥ 23 to 24.9 kg/m^2^, ○ Obese: BMI ≥ 25 kg/m^2^ *Waist circumference (WC)*: Waist circumference was measured to the nearest 0.1 cm using a nonstretchable dressmaker’s tape, with the subjects standing in the upright position. We measured at a level of the umbilicus, parallel to the floor. WC was classified as follows: - Caucasian cutoff^6^: ○ High: Male ≥ 94 cm: Female ≥ 80 cm. ○ Normal: Male < 94 cm: Female < 80 cm. - Asian cutoff^6^: ○ High: Male ≥80 cm: Female ≥ 78 cm. ○ Normal: Male < 80 cm: Female < 78 cm. *Hip circumference*: Hip circumference was measured by using a nonstretchable dressmaker’s tape at the largest circumference of the buttocks, parallel to the floor. *Waist-to-hip ratio (WHR*): was calculated by dividing WC (cm) by hip circumference (cm). Caucasian cutoff^7^: ○ High: Male ≥ 0.90; Female ≥ 0.85 ○ Normal: Male < 0.90; Female < 0.85 Asian^7^: ○ High: Male ≥ 0.90; Female ≥ 0.80 ○ Normal: Male < 0.90; Female < 0.80 *Waist-to-height ratio (WHtR)*: was calculated by dividing WC (cm) by height (cm). The general cutoff point for WHtR > 0.5 is considered high and WHtR ≤ 0.5 is considered normal; no difference is made between males and female.^10^ *Blood pressure measurement*: Blood pressure was measured three times, five minutes apart. The average from only the second and the third measurements was used, to obtain a more reliable estimate of an individual’s true blood pressure (eliminating the first reduces error due to physical activity or tension due to medical encounter) and to reduce the impact of measurement error on analyses.^3^ Hypertension (HTA) was defined according to WHO and MOH standard as blood pressure ≥140/90 mm Hg; isolated systolic hypertension was defined as a diastolic blood pressure less than 80 mm Hg and systolic blood pressure at 130 mm Hg or higher; isolated diastolic hypertension (IDH), an unrecognized subtype of hypertension, was defined as DBP more than 90 mm Hg and systolic BP less than 140 mm Hg or self-reported use of medication to treat HTA in the last two weeks. *Diabetes* was defined as having a hemoglobin A1c (HbA_1c_) level ≥6.5% or fasting blood glucose concentration (FBG) ≥7 mmol/L (≥ 126.0 mg/dl) after fasting 8 to 10 hours before the test or self-reported use of medication to treat diabetes in the last two weeks.^1^ The *dependent variables* (outcome variables) were diabetes and hypertension as defined above, while the *independent variables* were socio-demographic variables such as age, sex, region, ethnicity, and health/anthropometric outcomes such as BMI, waist circumference and hip circumference, and blood pressure.

## Statistical Analyses

Post-stratification weighting accounted for population distribution by districts, sex, and age groups from the National Population Census based on the 2020 National Census. Individual weighting was performed with the inverse of the probability of selection for each respondent, which was considered as the weight for the individual household. Receiver operating characteristics curve (ROC) analysis was performed to predict the sensitivity and specificity, of the studied measurements, using the Youden index (sensitivity + specificity - 1)^15^ to calculate the optimal cutoff of anthropometry indices. The area under the curve (AUC) from the ROC analysis with 95% CI was estimated for six anthropometric indices (BMI, WC, HC, WHR, WHtR, weight). The ability of the different indices to identify the risk of DM and HTA was compared according to Hanley and McNeil^16^ and the AUC prediction performance was classified as excellent (AUC ≥ 0.9), considerable (0.8 ≤ AUC <0.9), fair (0.7 ≤ AUC <0.8), poor (0.6 ≤ AUC <0.7), or failed (0.5 ≤ AUC <0.6).^17^ All statistical analyses were performed using Stata for Windows version 17.0.

## Results

Among 3242 participants, 1820 (58.22 %) were female; average age was 46 ± 14.65 years. Average weight, height, WC, and WHR were higher in males than females, but hip circumference and BMI were higher in women. There was no significant association between sex and the average obesity index (Annex 4).

Comparing BMI cutoff points used for Caucasians and Asians, the prevalence of obesity was >30% in both sexes using the Asian cutoff point, but only 4% (males) and 10% (females) using the Caucasian cutoff point. There was no difference between males and females using the Asian cutoff, but when the Caucasian cutoff was used, significantly more females were obese (*P* < .001) (Annex 5).

The mean weight of participants with DM only was 59.33 ± 12.38 kg, and for those with HTA only was 61.19 ± 12.19 kg. The mean BMI of individuals with both DM and HTA (26.07), HTA only (25.22), and DM only (24.35) was higher than that of healthy individuals (23.0). Similarly, WHtR was also higher among those with both DM and HTA (0.56), HTA only (0.54), and DM only (0.53) compared with the healthy group (0.50) (Table 1).

**Table 1.** Characteristics of Obesity Index and Health, DM, HTA, and Both DM and HTA.

	Healthy (n=915)				DM only^a^ (n= 444)				HTA only^b^ (n= 605)				Both DM and HTA (n= 134)			
	Mean	SD	95% CI		Mean	SD	95% CI		Mean	SD	95% CI		Mean	SD	95% CI	
Age	**42.98**	14.37	42.87	43.09	**51.11**	13.24	50.63	51.58	**54.80**	12.12	54.60	54.99	**57.64**	9.48	57.29	58.00
Weight	**55.92**	11.36	55.77	56.08	**59.33**	12.38	58.83	59.83	**61.19**	12.19	60.22	62.16	**63.58**	11.99	62.58	64.58
Height	**155.71**	7.64	155.61	155.82	**156.03**	6.77	155.78	156.28	**155.63**	7.52	154.99	156.28	**156.15**	6.15	155.72	156.58
HIP	**90.61**	8.77	90.49	90.73	**93.23**	10.02	92.79	93.66	**95.67**	9.57	94.91	96.43	**97.15**	10.21	96.23	98.06
WC	**76.89**	11.07	76.74	77.04	**82.30**	12.23	0.25	81.82	**84.98**	11.70	84.09	85.86	**87.71**	11.21	86.80	88.63
WHR	**0.85**	0.07	0.85	0.85	**0.88**	0.07	0.88	0.88	**0.89**	0.07	0.88	0.89	**0.90**	0.06	0.90	0.91
BMI	**23.01**	4.17	22.95	23.08	**24.35**	4.77	24.15	24.54	**25.22**	4.48	24.85	25.58	**26.07**	4.66	25.67	26.47
WHtR	**0.50**	0.07	0.50	0.50	**0.53**	0.53	0.52	0.53	**0.55**	0.08	0.54	0.55	**0.56**	0.07	0.56	0.57

DM only^a^: diabetic or history of treatment for diabetes in the last two weeks was defined as diabetes.

HTA only^b^: hypertension was defined as the having blood pressure ≥140/90 mm Hg and/or isolated systolic hypertension and/or isolated diastolic hypertension (IDH), or history of treatment for HTA in the last two weeks.

## Prediction of DM: Sensitivity and Specificity

**Figure 1.**
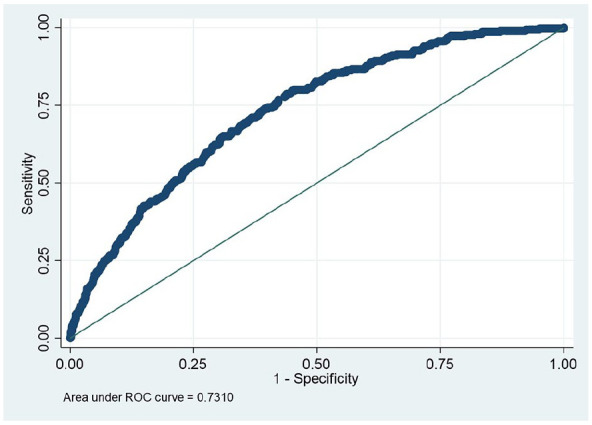
WHtR and DM (HbA_1c_), AUC = 0.73; 95% CI [0.70, 0.76].

The WHtR was found to have higher AUC in predicting DM, based on HbA_1c_, than did FBG or combined HbA_1c_ with FBG and treatment history. It was also better than BMI, with the AUC = 0.73; 95% CI [0.70, 0.76] for WHtR compared with AUC = 0.70; 95% CI [0.67, 0.73] for BMI (Figure 1).

## Using the WHtR to Predict DM by Sex, Age Group, and Ethnicity

The WHtR had better performance in predicting DM in males (AUC = 0.77; 95% CI [0.73, 0.82]) than in females (AUC = 0.69; 95% CI [0.65, 0.73]). It could also better predict DM at a younger age (18-34, AUC =0.90; 95% CI [0.82, 0.97]) compared with older age (AUC = 0.71; 95% CI [0.68, 0.75]) for 35- to 60-year-olds and (AUC = 0.66; 95% CI [0.60, 0.73]) for >60 years (Annex 6).

## Predicting HTA: Sensitivity and Specificity

**Figure 2.**
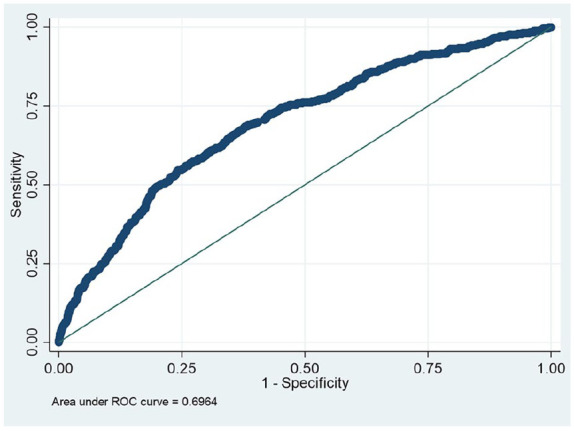
WHtR and HTA by measurement, AUC = 0.70; 95% CI [0.67, 0.72].

The WHtR was also better than other anthropometry indices at predicting risk of HTA, with AUC = 0.70; 95% CI [0.67, 0.72] (Figure 2).

## Using the WHtR to Predict HTA by Sex, Ethnicity, and Age Group

The WHtR is the better predictor of HTA in females (AUC = 0.72; 95% CI [0.69, 0.76]) compared with males (AUC = 0.67; 95% CI [0.63, 0.71]); in younger age (aged 18-34 years, AUC = 0.70; 95% CI [0.59, 0.81]) compared with older age (aged ≥60 years, AUC = 0.62; 95% CI [0.57, 0.68]) and in other ethnicities (Minority, AUC = 0.71; 95% CI [0.65, 0.78]) compared with Lao-Tai (Lao-Tai, AUC = 0.68; 95% CI [0.65, 0.72]) (Annex 7).

## Optimal Cutoff Points for WHtR to Identify DM and HTA

The optimal cutoff points for WHtR to identify DM in the Lao population (compared with those who have high HbA_1c_) is 0.5 (exact value = 0.497006). The AUC was 0.6723 (95% CI [0.64, 0.69]), indicating moderate discriminative ability.

The optimal cutoff point for WHtR to screen for HTA is also = 0.5 (exact value = 0.505992); the AUC of 0.6459 (95% CI [0.64, 0.69]) indicates fair discriminative ability.

## Optimal Cutoff Points for BMI to Identify DM by HbA_1c_ and HTA

The optimal BMI cutoff point for screening for DM in the Lao population, compared with patients having high HbA_1c_, is BMI >23.6 (exact value = 23.61164) with the AUC = 0.6573 (95% CI [0.63, 0.69]).

The optimal BMI cutoff point for screening for HTA in the Lao population is also BMI > 23.6 (exact value = 23.58833), with an AUC = 0.6307 (95% CI [0.61, 0.66]).

## Discussion

This study identified WHtR as the best anthropometric index with predictive power for DM and HTA in the Lao population. It is recommended for health workers to assess risks in the population and among patients, who are often unaware of their disease status.^1,3^

In Lao PDR, nutrition research and policy are inconsistent in the use of BMI as the obesity index. Some studies and national nutrition training guidelines use an international standard BMI (Caucasian),^14^ whereas others use an Asian BMI cutoff point.^1^ The standard BMI cutoff point recommended by WHO might give unrealistic nutritional status results, increasing the normal range and reducing overweight and obesity, compared with results using the Asian BMI cutoff. This was confirmed in our results and others from Asian countries such as Thailand^6^ and China.^8^ The optimal cutoff point used in Asian countries should be lower compared with the Caucasian BMI (BMI between 23 and 25) considered as a health risk, which corresponds to the Asia-Pacific cutoff point recommended by WHO. Therefore, we recommend using the Asia-Pacific cutoff point to identify the risk of NCDs in countries in this region which have not established their own BMI cutoff point. For Lao PDR, when BMI will be used, the cutoff point of BMI > 23.6 is considered to indicate high risk when screening for NCDs. Using the Caucasian cutoff resulted in missing too many people; patients with DM and/or HTA were classed as overweight or obese by Asian measures, but fell in the normal range using the international standard. We also found that the BMI of the healthy population was slightly higher compared with the Asian cutoff point, possibly because of differences in age, sex, and ethnic group. Other anthropometric indices used to identify DM, HTA, or both also resulted as high risk with the Asian cutoff point but normal with the Caucasian cutoff.

Although widely used to assess health, BMI has been criticized as a poor predictor of heart disease, DM and certain cancers, because it does not consider the distribution of body fat, muscle mass, and metabolic health.^18,19^ A person with a high BMI may have a lot of muscle mass and a low body fat, and may not be at risk for NCDs. Conversely, an individual with a “normal” BMI may have a high percentage or unhealthy distribution of body fat, which could increase their risk of NCDs. Studies reveal that BMI can differ significantly among different age, sexes, and ethnic groups^18^ based on the regions and demographics. Some ethnic groups are found to have a higher percentage of body fat and abdominal adipose tissue than Caucasians with the same BMI.^19^ Therefore, using specifically identified BMI cutoff was recommended.^20^

The highest performing anthropometric index to identify DM and HTA in our population was WHtR, with an optimal cutoff point of 0.5 to identify DM and HTA. The next best was WC, followed by WHR then BMI. Evidence from other countries has also suggested that WHtR was better than other anthropometric indices at identifying NCD risk factors.^8,9,21^ Yet, BMI and WC^10^ are still the most commonly used worldwide, also in Lao PDR.^1^ Measuring BMI also requires a weighing scale and a measuring board, which may be costly or unavailable, and challenging to transport to remote areas. Budgetary and other limitations restrict the placement of these tools in Lao health centers.^22^ Evidence from this and other studies is that optimal BMI cutoffs vary between sex and ethnic groups, which was not a problem with WHtR.^18,19^

The WC and WHtR have been proposed as alternative tools to measure abdominal adiposity and may be superior to BMI in terms of their performance in predicting NCD risk,^7^ as central obesity has been linked to a sedentary lifestyle, poor dietary choices, and stress. These behaviors can contribute to the accumulation of excess fat around the waistline, leading to central obesity and increased risk of NCDs. Central obesity may be a stronger predictor of mortality than other obesity indexes^23^ and measuring it is cost-effective, requiring less investment.^21^ The WC is suitable for screening NCDs in a large population and in remote areas. However, the international standard cutoff points for BMI and WC are not recommended; any anthropometric measure is associated with ethnic- and population-specific disease risks.^7^

The WHtR has been reported to be a more accurate predictor of cardiovascular risk in comparison with WC and other indices,^10,21,24^ specifically in populations with a higher risk of metabolic disorders and type 2 DM, as often found in urban areas. Our study also found that WHtR is an effective predictor of high HbA_1C_ levels in young patients with DM (18-34 years old). This makes WHtR a valuable screening tool for NCDs. Early identification can reduce complications and treatment costs, which are significant economic burdens due to the chronic nature of these diseases and long-term need for supportive medications as the primary intervention.

The WHtR considers the height, which may provide a more precise reflection of overall body size and fat distribution than WC alone. Adult height is considered to be relatively stable over time, little influenced by nutrition or health.^25^ It can also be a predictor for NCDs, with taller people being at increased risk of certain types of cancer^26^ and shorter people at increased risk of cardiovascular disease and type 2 DM.^27,28^

We found that the average WHtR in our study and the optimal cutoff, WHtR = 0.5 (0.497006), were in line with those suggested by WHO and various studies as globally applicable in screening for cardiovascular and metabolic risk^7-9^ with the ability to screen other health indicators and clinical markers such as the albumin excretion rate and coronary artery disease.^29,30^ We did not find any significant difference between the majority and minority ethnic groups in Lao PDR, which is consistent with results of a meta-analysis, that WHtR was strongly associated with DM for both Asian and non-Asian populations. It can also provide a clear call-to-action message, like “A healthy waist is less than half your height, so get moving and eat right.” Such a message is simple and can be used universally, also in an NCDs prevention campaign where obesity and NCDs are on the rise, as in Lao PDR.

The technique that we used to obtain WC in this study was to measure parallel to the floor, at the level of the umbilicus, rather than at the narrowest point between the lower costal border and the top of the iliac crest, as recommended by the WHO.^7^ There is evidence that measuring the WC at the umbilicus^23^ provides higher performance in detecting body fat, especially in women, leading to a recommendation to standardize the measurement location; it proved easy to find the place for measurement in practice. We also found that WHtR was effective in predicting health outcomes regardless of sex; however, its performance was slightly higher in women for HTA and in males for DM. Further studies are recommended to confirm whether measurement of WC at the umbilicus provides higher sensitivity in the Lao population than measuring at the iliac crest, as recommended by WHO.

In the literature, it is not clear whether WHtR is more effective in predictive performance in males or females; the reported results do not reach a consensus. The slight difference in sensitivity that we found may depend on factors such as age and health. However, a systematic review and meta-analysis found that the predictive ability of WHtR did not differ significantly between men and women, suggesting that WHtR may be equally effective in both sexes.^10,11^

## Limitations

This was a cross-sectional study with biological examination at one time point. It did not account for other factors related to increasing obesity or possible risk behaviors related to DM and HTA. The optimal cutoff point was determined by optimizing both sensitivity and specificity to compare the performance of different anthropometric indices. In clinical settings, such as assessing risk for NCDs, the cutoff point could be adjusted to increase the sensitivity of the screening test, thereby enhancing the ability to identify potentially important health outcomes.

## Recommendations

Waist-to-height ratio is a simple, cost-effective, affordable, portable, and effective way to measure central obesity and is a better predictor of DM, HTA, cardiovascular disease, and other NCDs risk, than BMI. Our results are supported by reports in the literature that recommend using the WHtR as indicator for health promotion and prevention campaigns. The WHtR can also be used as first-stage screening tool for NCDs. The cutoff point of 0.5 was easy to remember and can be included in health promotion slogans, which should be created in cooperation with community members and patients to ensure relevance. The WHtR can also be useful as a target message for NCD patients to monitor their waistline along with regular treatment and changing lifestyles, particularly in urban areas with their ongoing food and lifestyle transition but where people also start to pay more attention to their own health. The index can be used together with standard health examinations during health checks or physical examinations to monitor risk and start to reduce morbidity and mortality. We do recommend using WHtR as a screening tool and in public awareness campaigns for DM and, HTA, in both urban and rural remote areas or in fieldwork under unfavorable conditions.

## Conclusion

The research agenda for nutrition, prepared with participation by actors at all levels, led to this research, the results of which in turn will inform planning of health promotion activities, which should also be planned in a participatory way. The WHtR could serve as screening indices for NCDs prevention and control, and can be used in urban areas, with a high prevalence of DM and HTA, and in rural areas, in fieldwork under difficult conditions. We can use the WHtR for health prevention and promotion in urban areas along with appropriate advice about changing lifestyle factors. The WHtR can work both as a public health and a clinical screening tool for monitoring the DM and HTA in Lao PDR, using the optimal cutoff point of 0.5 for both DM and HTA in both sexes and all ethnicities.

## Supplemental Material

sj-docx-1-aph-10.1177_10105395241295573 – Supplemental material for Waist-to-Height Ratio as a Key Predictor for Diabetes and Hypertension in Lao PDR National Health Survey Supplemental material, sj-docx-1-aph-10.1177_10105395241295573 for Waist-to-Height Ratio as a Key Predictor for Diabetes and Hypertension in Lao PDR National Health Survey by Kethmany Ratsavong, D. R. Essink, Manithong Vonglokham, Sengchanh Kounnavong, Somphou Sayasone, Wichai Aekplakorn, Suchin Worawichawong and E. P. Wright in Asia Pacific Journal of Public Health

## References

[1] Vonglokham M Kounnavong S Sychareun V Pengpid S Peltzer K. Prevalence and social and health determinants of pre-diabetes and diabetes among adults in Laos: a cross-sectional national population-based survey, 2013. Trop Med Int Health. 2019;24(1):65-72. doi:10.1111/tmi.13164 30303580

[2] Laos. Institute for health metrics and evaluation. Published September 9, 2015. Accessed April 18, 2023. https://www.healthdata.org/laos

[3] Pengpid S Vonglokham M Kounnavong S Sychareun V Peltzer K. The prevalence, awareness, treatment, and control of hypertension among adults: the first cross-sectional national population-based survey in Laos. Vasc Health Risk Manag. 2019;15:27-33. doi:10.2147/VHRM.S199178 30881005 PMC6398413

[4] Tiptaradol S Aekplakorn W. Prevalence, awareness, treatment and control of coexistence of diabetes and hypertension in Thai population. Int J Hypertens. 2012;2012:386453. doi:10.1155/2012/386453 22888406 PMC3408662

[5] Piqueras P Ballester A Durá-Gil JV Martinez-Hervas S Redón J Real JT. Anthropometric Indicators as a tool for diagnosis of obesity and other health risk factors: a literature review. Front Psychol. 2021;12. doi:10.3389/fpsyg.2021.631179 PMC8299753 34305707

[6] Samsen M Hanchaiphiboolkul S Puthkhao P Tantirittisak T Towanabut S . Appropriate body mass index and waist circumference cutoffs for middle and older age group in Thailand: data of 19,621 participants from Thai epidemiologic stroke (TES) study. J Med Assoc Thai. 2012;95(9):1156-1166. 23140032

[7] WHO. Waist circumference and waist-hip ratio: report of a WHO expert consultation. Accessed December 13, 2022. https://www.who.int/publications-detail-redirect/9789241501491

[8] Tseng CH Chong CK Chan TT , et al. Optimal anthropometric factor cutoffs for hyperglycemia, hypertension and dyslipidemia for the Taiwanese population. Atherosclerosis. 2010;210(2): 585-589. doi:10.1016/j.atherosclerosis.2009.12.015 20053403

[9] Browning LM Hsieh SD Ashwell M. A systematic review of waist-to-height ratio as a screening tool for the prediction of cardiovascular disease and diabetes: 0·5 could be a suitable global boundary value. Nutr Res Rev. 2010;23(2):247-269. doi:10.1017/S0954422410000144 20819243

[10] Ashwell M Gunn P Gibson S. Waist-to-height ratio is a better screening tool than waist circumference and BMI for adult cardiometabolic risk factors: systematic review and meta-analysis: waist-to-height ratio as a screening tool. Obes Rev. 2012;13(3):275-286. doi:10.1111/j.1467-789X.2011.00952.x 22106927

[11] Siwarom S Pirojsakul K Aekplakorn W , et al. Waist-to-height ratio is a good predictor of metabolic syndrome in adolescents: a report from the Thai national health examination survey V, 2014. Asia Pac J Public Health. 2022;34(1):36-43. doi:10.1177/10105395211046474 34590882

[12] Essink DR Ratsavong K Bally E , et al. Developing a national health research agenda for Lao PDR: prioritising the research needs of stakeholders. Glob Health Action. 2020;13(suppl 2): 1777000. doi:10.1080/16549716.2020.1777000 PMC7480602 32741341

[13] Wilson H Cummings J Rasprasith S Stadler D. Evaluation of a nutrition-risk screening tool in Lao PDR: identifying malnutrition in a low-resource clinical setting. Clin Nutr ESPEN. 2020;38:99-110. doi:10.1016/j.clnesp.2020.05.023 32690186

[14] Department of Hygiene and Health Promotion Ministry of Health of Lao PDR. Operational manual nutrition sentinel surveillance in Lao PDR. Accessed September 14, 2024. https://ag.purdue.edu/department/ipia/anrcb/_docs/resources/nutrition-sentinel-surveillance-in-lao-pdr-2023-min.pdf

[15] Schisterman EF Faraggi D Reiser B Hu J. Youden Index and the optimal threshold for markers with mass at zero. Stat Med. 2008;27(2):297-315. doi:10.1002/sim.2993 17624866 PMC2749250

[16] Hanley JA McNeil BJ. The meaning and use of the area under a receiver operating characteristic (ROC) curve. Radiology. 1982;143(1):29-36. doi:10.1148/radiology.143.1.7063747 7063747

[17] Çorbacıoğlu ŞK Aksel G . Receiver operating characteristic curve analysis in diagnostic accuracy studies: a guide to interpreting the area under the curve value. Turk J Emerg Med. 2023;23(4):195-198. doi:10.4103/tjem.tjem_182_23 38024184 PMC10664195

[18] Adab. Is BMI the best measure of obesity? Published 2018. Accessed April 18, 2023. https://www.bmj.com/content/bmj/360/bmj.k1274.full.pdf 10.1136/bmj.k1274 29599212

[19] Rush EC Freitas I Plank LD. Body size, body composition and fat distribution: comparative analysis of European, Maori, Pacific Island and Asian Indian adults. Br J Nutr. 2009;102(4): 632-641. doi:10.1017/S0007114508207221 19203416

[20] Chen X Wang Y. Commentary: optimal body mass index cut points. Int J Epidemiol. 2010;39(4):1045-1047. doi:10.1093/ije/dyq081 20488882 PMC2929354

[21] Ashwell M Gibson S. Waist-to-height ratio as an indicator of “early health risk”: simpler and more predictive than using a “matrix” based on BMI and waist circumference. BMJ Open. 2016;6(3):e010159. doi:10.1136/bmjopen-2015-010159 PMC4800150 26975935

[22] Phommachanh S Essink DR Wright EP Broerse JEW Mayxay M. Do health care providers give sufficient information and good counseling during ante-natal care in Lao PDR?: an observational study. BMC Health Serv Res. 2019;19(1):449. doi:10.1186/s12913-019-4258-z 31272432 PMC6611023

[23] Kagawa M Byrne NM Hills AP. Comparison of body fat estimation using waist: height ratio using different “waist” measurements in Australian adults. Br J Nutr. 2008;100(5):1135-1141. doi:10.1017/S0007114508966095 18341757

[24] Ke JF Wang JW Lu JX Zhang ZH Liu Y Li LX. Waist-to-height ratio has a stronger association with cardiovascular risks than waist circumference, waist-hip ratio and body mass index in type 2 diabetes. Diabetes Res Clin Pract. 2022;183:109151. doi:10.1016/j.diabres.2021.109151 34863718

[25] Silventoinen K Sammalisto S Perola M , et al. Heritability of adult body height: a comparative study of twin cohorts in eight countries. Twin Res off J Int Soc Twin Stud. 2003;6(5):399-408. doi:10.1375/136905203770326402 14624724

[26] Green J Cairns BJ Casabonne D , et al. Height and cancer incidence in the Million Women Study: prospective cohort, and meta-analysis of prospective studies of height and total cancer risk. Lancet Oncol. 2011;12(8):785-794. doi:10.1016/S1470-2045(11)70154-1 21782509 PMC3148429

[27] Fahim O Naghshi S Khademi Z Esmaillzadeh A. Association of adult height with cardiovascular mortality: a systematic review and meta-analysis of cohort studies. Int J Clin Pract. 2022;2022:6959359. doi:10.1155/2022/6959359 36349059 PMC9629919

[28] Song W Hu Y Yuan J , et al. Gender differences between the phenotype of short stature and the risk of diabetes mellitus in Chinese adults: a population-based cohort study. Front Endocrinol. 2022;13:869225. doi:10.3389/fendo.2022.869225 PMC9016201 35450422

[29] Tseng CH. Waist-to-height ratio is independently and better associated with urinary albumin excretion rate than waist circumference or waist-to-hip ratio in Chinese adult type 2 diabetic women but not men. Diabetes Care. 2005;28(9):2249-2251. doi:10.2337/diacare.28.9.2249 16123501

[30] Tseng CH. Waist-to-height ratio and coronary artery disease in Taiwanese type 2 diabetic patients. Obesity. 2008;16(12):2754-2759. doi:10.1038/oby.2008.430 18927550

