# Supplementary material for: Waist-to-Height Ratio as a Key Predictor for Diabetes and Hypertension in Lao PDR National Health Survey
Source: Asia Pac J Public Health. 2024 Oct 31;37(1):35–42. doi: 10.1177/10105395241295573 (PMC11894911; doi:10.1177/10105395241295573)
Supplement: sj-docx-1-aph-10.1177_10105395241295573 – Supplemental material for Waist-to-Height Ratio as a Key Predictor for Diabetes and Hypertension in Lao PDR National Health Survey [file sj-docx-1-aph-10.1177_10105395241295573.docx]

**Supplementary Material:**

**Annex 1: Sample Size calculation**

A representative sample of adults in Lao PDR who are 18 years old or older will be selected using a multi-stage cluster sampling method. The sample size for the study will be 3,227 individuals, which will be rounded up to 3,240 to facilitate logistical considerations

Sample size has been estimated using the formula:

n= Z^2^ P(1-P)

e^2^

Where n= sample size, Z= level of confidence, P= baseline level of the indicators and e= margin of error.

Given the estimated prevalence risk factors, P= 0.3 (prevalence of currently smoke tobacco), Z= 1.96 (at 95% confidence interval), e= 0.05, the initial estimated sample size would be:

n= 1.96^2^0.5(1-0.5) = 323

0.05^2^

Adjusting for:

Design effect for complex sample design 1.50 (multiply)

Strata for reporting Age-sex estimates 6 (multiply)

Anticipated non-response= 10% (divide by 0.9)

The estimated required sample size is therefore:

n=323* 1.5* 4= 3,227

0.9

Based on the estimation, a minimum size of 3,227 participants is required, which will be rounded up to 3,240 for logistical convenience.

It is planned to approximately 1,080 individuals for venous blood sample collection.

To ensure the representativeness of the sample at the national level, a multi-stage sampling technique were employed, involving the selection of enumeration areas, households, and eligible participants in three sequential stages

**Annex 2:** Sampling procedures

A multi-stage cluster sampling method will be used to select a nationally representative eligible sample participant in Lao PDR using a three-stage sampling procedure as following steps:

1. Random Selected the probability proportional to size (PPS) by using a sampling method applied to select the study villages (i.e., the primary sampling unit (PSU)) based on the village's list in each of 18 provinces across the country based on the 2015 National Census, the Lao statistical Bureau of villages sampling frame for the population aged equal to or over 18 years which suggested to select 165 villages (PSUs) from the total number of all villages.

2. The simple random technique was used to select 20 households in each village as the secondary sampling units (SSUs) using the list of households available in the village. A total of 20 households were recruited from each target selected village.

3. One eligible participant aged 18 years or older from each selected household was randomly selected using the Kish sampling method by ranked in order of decreasing age, starting with males than females, then randomly selected using the automated program for Kish selection in the digital device.

**Annex 3: Field and laboratory procedures**

**Fieldwork preparation**

The survey team comprised 6 teams including 1 supervisor (planning and checking the completeness of questionnaires, and undertaking some interviews/measurement), 3 field data collectors (for STEP 1 and STEP 2, and STEP 3), and 1 driver (transport). Each team included one or two survey administrators with some medical background to assist with STEP 3 measures and a laboratory technician for taking blood samples. A village volunteer and village authority assisted each interviewer to look for selected households and support the whole team in surveying the village.

**Logistics of fieldwork:**

Before going to the sample village, the team leader had to contact and coordinated with the local authorities for prepared a list of households in the village for the survey team and announced the survey. Specified the province, district, village, and household ID for the team to use during the interviews and blood tests. The preparation by the team leader has been almost important in facilitating the fieldwork team and maintaining oneness within the team.

**Laboratory procedures**

In each study participant, a blood sample of 10 ml was collected and aliquoted into three types of vacuum trainer tubes. The clot blood red tube was aliquoted about 4 ml and centrifuged in the field after 30 minutes of sample collection. The centrifugation was set at 3000rpm for 10 minutes. After centrifuge, about 1.3 ml of serum was aliquot into a prepared serum and kept in a cool box for lipid profile test. EDTA and NaF tubes, each aliquoted 3 ml of blood samples and gently checked 8-10 times before keeping them in a cool box and the centrifuge was done at the Roche center. EDTA blood sample was subjected to a test for HbA1c and NaF for blood glucose. All blood samples were sent to the Roche laboratory center at Military 103 Hospital and analyzed on the day of sample collection. The sample analysis used the Cobos Integra 400 plus, Chemistry analyzer (Roche company, Germany).

**Anthropometry Procedures:**

Weight, height, waist, and hip circumference were collected by trained anthropometrists in each participant. A digital floor scale was used with 20g and 50g precision . A height measurement tool for adults was used with 0.5 cm precision. All measurements are collected in duplicate; if measurements differ by greater than 0.1 kg (weight), or 0.5 cm (length, height, the measurement is repeated a third time.

**Data quality control**

**Quality checks during fieldwork**

Each member of the survey team entered data using digital devices to record the respondent’s answers to the STEP 1 interview and the physical and biochemical results from STEP 2 and 3. A storage device card was fitted in every device to ensure a backup copy of data was stored in case of any device failures. No additional data entry was required as all data was entered at the time of the interview on digital devices.

Data from digital devices were sent to Lao TPHI institute every day via the internet then the data was checked. All data were downloaded into a single master database following the completion of the fieldwork. Data cleaning and weighting were undertaken before data analysis, following guidance provided by the survey manual. This included checking ranges and combinations of variables, detecting and handling missing data, and detecting and handling outliers.

| **BMI class** | **BMI cutoff point** | | | | | | | | | | | |
| --- | --- | --- | --- | --- | --- | --- | --- | --- | --- | --- | --- | --- |
|  | **Caucasian*, n(%)** | | | | | | **Asian** , n(%)** | | | | | |
|  | **Male** | | **Female** |  | **Total** |  | **Male** | | **Female** | | **Total** | |
|  | **(N)** | **%** | **(N)** | **%** | **(N)** | **%** | **(N)** | **%** | **(N)** | **%** | **(N)** | **%** |
| Underweight | (152) | 9.522 | (195) | 10.14 | (347) | 9.881 | (152) | 9.52 | (195) | 10.14 | (347) | 9.88 |
| Normal | (926) | **61.67** | (1016) | **54.12** | (1942) | 57.3 | (712) | 45.88 | (709) | 36.67 | (1421) | 40.55 |
| Overweight | (265) | **24.37** | (399) | **25.7** | (664) | **25.14** | (214) | 15.78 | (307) | 17.45 | (521) | 16.75 |
| Obese | (48) | **4.44** | (140) | **10.03** | (188) | 7.675 | (313) | 28.81 | (539) | 35.74 | (852) | 32.82 |

**Annex 4:** **The average obesity index and sex** (supplementary table number 1).

|  | Male | | | | Female | | | |
| --- | --- | --- | --- | --- | --- | --- | --- | --- |
| Obesity index | Mean | SD | CI95% Min | CI95% Max | Mean | SD | CI95% Min | CI95% Max |
| Mean Age  (45.86 ± 14.65) | **47.18** | 15.33 | 47.03 | 47.33 | 44.92 | 14.10 | 44.77 | 45.07 |
| Average Weight | **59.80** | 11.77 | 59.59 | 60.01 | 55.20 | 11.20 | 55.01 | 55.40 |
| Average Height | **160.96** | 6.65 | 160.85 | 161.07 | 151.90 | 5.49 | 151.82 | 151.98 |
| Average HIP | 90.50 | 7.91 | 90.36 | 90.65 | **92.62** | 9.74 | 92.45 | 92.79 |
| Average WC | **79.55** | 11.48 | 79.35 | 79.75 | 78.31 | 11.66 | 78.13 | 78.50 |
| Average WHR | **0.88** | 0.07 | 0.87 | 0.88 | 0.84 | 0.06 | 0.84 | 0.84 |
| Average WHtR | 0.49 | 0.07 | 0.49 | 0.50 | **0.52** | 0.08 | 0.51 | 0.52 |
| Average BMI | 23.01 | 3.90 | 22.94 | 23.08 | **23.89** | 4.54 | 23.81 | 23.97 |

**Annex 5:** Comparison of BMI cutoff points between Caucasian and Asian classifications (supplementary table number 2)**.**

**Annex 6:** Sensitivity, speciﬁcity, predictive value, and distance in the receiver operating characteristic (ROC) curve of WHtR, WHR, WC, BMI, HC, and weight to identify subjects with diabetes diagnosed by HbA1c **(**supplementary figure number 1)

| Fi WHtR and DM (HbA1c) by sex  1= male; 2 = female **** | W WHtR and DM (HbA1c) by age group  1 = 18-34; 2 = 35-60; 3 = >=60-year-old **** | โร WHtR and DM (HbA1c) by ethnic group  0 = minority ethnic; 1 = Lao-Tai **** |
| --- | --- | --- |
|  WHR and DM (HbA1c), ROC = 0.70 |  WC and DM (HbA1c), ROC = 0.72 |  BMI and DM (HbA1c), ROC= 0.70 |
| HC and DM (HbA1c), ROC= 0.66 |  Weigh and DM (HbA1c), ROC=0.66 |  |

| WHtR and HTA by sex  1= male; 2 = female | WHtR and HTA by age group  1 = 18-34; 2 = 35-60; 3 = >=60-year-old | WHtR and HTA by ethnic group  0 = minority ethnic; 1 = Lao-Tai |
| --- | --- | --- |
| WHR and HTA by measurement,  ROC = 0.66 | WC and HTA by measurement,  ROC = 0.69 | BMI and HTA by measurement,  ROC = 0.65 |
| HC and HTA by measurement,  ROC = 0.66  | Weigh and HTA by measurement, ROC=0.63 |  |

**Annex 7:** Sensitivity, speciﬁcity, predictive value, and distance in the receiver operating characteristic (ROC) curve of WHtR, WHR, WC, BMI, HC, and weight to identify subjects with DM by measurement and sex, ethnic and age group. **(**supplementary figure number 2)

**Annex 8:** The anthropometry indices measurement and other DM RBP tests:

**HbA1C** (supplementary table number 3)

|  |  | ROC |  | Asymptotic normal |
| --- | --- | --- | --- | --- |
|  | Obs | area | Std. err. | [95% conf. interval] |
| WHtR | 2,594 | **0.731** | 0.0146 | 0.70247 0.75955 |
| WC | 2,594 | 0.7187 | 0.0151 | 0.68900 0.74837 |
| WHR | 2,594 | 0.7022 | 0.0157 | 0.67132 0.73305 |
| BMI | 2,594 | 0.6991 | 0.016 | 0.66782 0.73036 |
| WHtR  (optmal cut-off for HbA1C ) | 2,594 | 0.6743 | 0.0129 | 0.64894 0.69962 |
| WHtR (Standard cut-off) | 2,594 | 0.6723 | 0.0133 | 0.64633 0.69836 |
| WHtR (optmal cut-off for RBP) | 2,594 | 0.6712 | 0.0137 | 0.64442 0.69802 |
| BMI (Asian cut-off) | 2,594 | 0.6703 | 0.0156 | 0.63963 0.70096 |
| WC (Asian cut-off) | 2,594 | 0.6683 | 0.0138 | 0.64134 0.69531 |
| Weight | 2,594 | 0.6649 | 0.0167 | 0.63207 0.69769 |
| WHR (Caucasian cut-off) | 2,594 | 0.6606 | 0.0142 | 0.63279 0.68840 |
| BMI (Caucasian cut-off) | 2,594 | 0.6603 | 0.0159 | 0.62916 0.69137 |
| WHR (Asian cut-off) | 2,594 | 0.6603 | 0.0119 | 0.63696 0.68358 |
| Hip Circumference | 2,594 | 0.6594 | 0.0174 | 0.62538 0.69340 |
| WC (Caucasian cut-off) | 2,594 | 0.6405 | 0.0151 | 0.61092 0.67009 |
|  |  |  |  |  |

**HbA1C or Fasting blood glucose concentration** (supplementary table number 4)

|  |  | ROC |  | Asymptotic normal | |
| --- | --- | --- | --- | --- | --- |
|  | Obs | area | Std. err. | [95% conf. interval] | |
| WHtR | 2,594 | **0.6866** | 0.015 | 0.6571 | 0.71606 |
| WC | 2,594 | 0.6813 | 0.0153 | 0.65135 | 0.7112 |
| WHR | 2,594 | 0.6772 | 0.0152 | 0.64735 | 0.70704 |
| BMI | 2,594 | 0.6565 | 0.0158 | 0.62548 | 0.68751 |
| WHtR (optmal cut-off for HbA1C) | 2,594 | 0.6447 | 0.0128 | 0.61953 | 0.66982 |
| WC (Asian cut-off) | 2,594 | 0.6423 | 0.0133 | 0.61625 | 0.6684 |
| WHtR (Standard cut-off) | 2,594 | 0.6415 | 0.0131 | 0.61588 | 0.66712 |
| WHR (Caucasian cut-off) | 2,594 | 0.6409 | 0.0135 | 0.61453 | 0.66736 |
| WHtR (optmal cut-off for RBP) | 2,594 | 0.6398 | 0.0133 | 0.6137 | 0.66596 |
| Weight | 2,594 | 0.6372 | 0.0159 | 0.60597 | 0.66848 |
| BMI (Asian cut-off) | 2,594 | 0.6366 | 0.0151 | 0.60714 | 0.66614 |
| BMI (Caucasian cut-off) | 2,594 | 0.6334 | 0.0148 | 0.60435 | 0.66246 |
| WHR (Asian cut-off) | 2,594 | 0.6316 | 0.0121 | 0.60789 | 0.65536 |
| Hip Circumference | 2,594 | 0.6224 | 0.0167 | 0.58964 | 0.65517 |
| WC (Caucasian cut-off) | 2,594 | 0.6145 | 0.0138 | 0.58746 | 0.64156 |
|  |  |  |  |  |  |

**Fasting blood glucose concentration only** (supplementary table number 5)

|  |  | ROC |  | Asymptotic normal | |
| --- | --- | --- | --- | --- | --- |
|  | Obs | area | Std. err. | [95% conf. interval] | |
| WHR | 2,596 | **0.662** | 0.0189 | 0.62507 | 0.69896 |
| WC | 2,596 | 0.6548 | 0.0191 | 0.61744 | 0.69213 |
| WHtR | 2,596 | 0.6503 | 0.0191 | 0.6129 | 0.68774 |
| WHR (Caucasian cut-off) | 2,596 | 0.63 | 0.0164 | 0.59785 | 0.66221 |
| WC (Asian cut-off) | 2,596 | 0.624 | 0.0163 | 0.59192 | 0.65599 |
| WHtR (optmal cut-off for HbA1C) | 2,596 | 0.6193 | 0.016 | 0.58806 | 0.6506 |
| WHtR (Standard cut-off) | 2,596 | 0.619 | 0.0162 | 0.58731 | 0.65068 |
| WHtR (optmal cut-off for RBP) | 2,596 | 0.6137 | 0.0165 | 0.5813 | 0.64605 |
| WHR (Asian cut-off) | 2,596 | 0.6111 | 0.015 | 0.58164 | 0.6405 |
| Weight | 2,596 | 0.6098 | 0.0198 | 0.57102 | 0.64853 |
| BMI | 2,596 | 0.6079 | 0.02 | 0.56873 | 0.64708 |
| BMI (Caucasian cut-off) | 2,596 | 0.6027 | 0.0182 | 0.56699 | 0.63834 |
| WC (Caucasian cut-off) | 2,596 | 0.6006 | 0.0169 | 0.56755 | 0.63365 |
| BMI (Asian cut-off) | 2,596 | 0.5999 | 0.0188 | 0.5631 | 0.63677 |
| Hip Circumference | 2,596 | 0.5953 | 0.0203 | 0.55553 | 0.6351 |

**HbA1C & Fasting blood glucose concentration & report DM treatment used** (supplementary table number 6)

|  |  | ROC |  | Asymptotic normal | |
| --- | --- | --- | --- | --- | --- |
|  | Obs | area | Std. err. | [95% conf. interval] | |
|  |  |  |  |  |  |
| WHtR | 2,597 | **0.6828** | 0.0143 | 0.65473 | 0.71077 |
| WC | 2,597 | 0.6759 | 0.0146 | 0.64724 | 0.70465 |
| WHR | 2,597 | 0.6723 | 0.0146 | 0.64362 | 0.70089 |
| BMI | 2,597 | 0.6528 | 0.015 | 0.62348 | 0.68221 |
| WHtR (optmal cut-off for RBP) | 2,597 | 0.6438 | 0.0123 | 0.61965 | 0.66798 |
| WHR (Caucasian cut-off) | 2,597 | 0.6428 | 0.0129 | 0.6176 | 0.66806 |
| WHtR (Standard cut-off) | 2,597 | 0.639 | 0.0128 | 0.614 | 0.66408 |
| WHtR (optmal cut-off for HbA1C) | 2,597 | 0.639 | 0.0126 | 0.61438 | 0.66368 |
| WC (Asian cut-off) | 2,597 | 0.637 | 0.0128 | 0.61191 | 0.66215 |
| Weight | 2,597 | 0.6314 | 0.0153 | 0.60138 | 0.66145 |
| BMI (Asian cut-off) | 2,597 | 0.6313 | 0.0144 | 0.60318 | 0.65944 |
| WHR (Asian cut-off) | 2,597 | 0.6302 | 0.0117 | 0.60728 | 0.65311 |
| BMI (Caucasian cut-off) | 2,597 | 0.6271 | 0.014 | 0.59962 | 0.65455 |
| Hip Circumference | 2,597 | 0.6212 | 0.0159 | 0.59004 | 0.65236 |
| WC (Caucasian cut-off) | 2,597 | 0.61 | 0.0131 | 0.5843 | 0.63568 |

**Raise Blood Pressure (**supplementary table number 7)

|  |  | ROC |  | Asymptotic normal | |
| --- | --- | --- | --- | --- | --- |
|  | Obs | area | Std. err. | [95% conf. interval] | |
| WHtR | 3,138 | **0.6964** | 0.0139 | 0.66924 | 0.72366 |
| WC | 3,138 | 0.6935 | 0.0139 | 0.66625 | 0.72085 |
| WHR | 3,138 | 0.666 | 0.0147 | 0.63712 | 0.69491 |
| Hip Circumference | 3,138 | 0.6606 | 0.0143 | 0.63252 | 0.6887 |
| BMI | 3,138 | 0.6539 | 0.0146 | 0.6252 | 0.68257 |
| WHtR (optmal cut-off for RBP ) | 3,138 | 0.6537 | 0.0122 | 0.6297 | 0.67761 |
| WC (Asian cut-off) | 3,138 | 0.6481 | 0.0123 | 0.62396 | 0.67227 |
| WHtR (Standard cut-off) | 3,138 | 0.6473 | 0.0121 | 0.62357 | 0.67104 |
| WHtR (optmal cut-off for HbA1C ) | 3,138 | 0.6459 | 0.012 | 0.62245 | 0.6694 |
| BMI (Asian cut-off) | 3,138 | 0.6411 | 0.0141 | 0.61349 | 0.66869 |
| WHR (Caucasian cut-off) | 3,138 | 0.635 | 0.0126 | 0.61033 | 0.65964 |
| Weight | 3,138 | 0.6325 | 0.0148 | 0.60355 | 0.66143 |
| WC (Caucasian cut-off) | 3,138 | 0.6232 | 0.0127 | 0.59819 | 0.64813 |
| BMI (Caucasian cut-off) | 3,138 | 0.6217 | 0.0139 | 0.5945 | 0.64899 |
| WHR (Asian cut-off) | 3,138 | 0.6072 | 0.0118 | 0.58416 | 0.63026 |

**Raise Blood Pressure + treatment** (supplementary table number 8)

|  |  | ROC |  | Asymptotic normal | |
| --- | --- | --- | --- | --- | --- |
|  | Obs | area | Std. err. | [95% conf. interval] | |
|  |  |  |  |  |  |
| WHtR | 3,138 | **0.7027** | 0.0125 | 0.67809 | 0.72725 |
| WC | 3,138 | 0.696 | 0.0126 | 0.67127 | 0.72081 |
| WHR | 3,138 | 0.6692 | 0.0132 | 0.64338 | 0.69497 |
| Hip Circumference | 3,138 | 0.6614 | 0.0131 | 0.63571 | 0.68712 |
| WHtR (Standard cut-off) | 3,138 | 0.66 | 0.011 | 0.63847 | 0.68156 |
| BMI | 3,138 | 0.652 | 0.0132 | 0.6261 | 0.67791 |
| WHtR (optmal cut-off for HbA1C) | 3,138 | 0.6514 | 0.0109 | 0.62993 | 0.67281 |
| WHtR (optmal cut-off for RBP) | 3,138 | 0.6501 | 0.0108 | 0.62891 | 0.67137 |
| WC (Asian cut-off) | 3,138 | 0.6492 | 0.0111 | 0.62741 | 0.67107 |
| WHR (Caucasian cut-off) | 3,138 | 0.6459 | 0.0112 | 0.62392 | 0.66798 |
| BMI (Asian cut-off) | 3,138 | 0.64 | 0.0126 | 0.61533 | 0.66473 |
| WC (Caucasian cut-off) | 3,138 | 0.6282 | 0.0113 | 0.60596 | 0.65036 |
| Weight | 3,138 | 0.6255 | 0.0134 | 0.59927 | 0.65172 |
| BMI (Caucasian cut-off) | 3,138 | 0.6252 | 0.0123 | 0.60108 | 0.64926 |
| WHR (Asian cut-off) | 3,138 | 0.6143 | 0.0106 | 0.5935 | 0.63502 |
